# Transesophageal echocardiography: Revolutionizing perioperative cardiac care

**DOI:** 10.17305/bb.2024.10847

**Published:** 2024-08-15

**Authors:** Jiuqing Liang, Xiaoyu Ma, Genqiang Liang

**Affiliations:** 1 Department of Anesthesiology, Zhuhai People’s Hospital (Zhuhai Hospital Affiliated with Jinan University), Zhuhai, China

**Keywords:** Cardiovascular diseases (CVDs), perioperative management, transesophageal echocardiography (TEE), artificial intelligence (AI), standardized training, multidisciplinary collaboration

## Abstract

Cardiovascular diseases (CVDs) are a major challenge in global health. Despite significant advances in treatment and management, the incidence and mortality rates of CVDs have been rising in recent years, particularly in the United States. With continuous advancements in medical technology, perioperative transesophageal echocardiography (TEE) has become a key technology in cardiac surgery, enhancing surgical success rates and patient safety. The application of TEE spans preoperative planning, intraoperative monitoring, and postoperative evaluation, especially in complex procedures, such as mitral valve repair and aortic valve replacement, where it plays an indispensable role. Simultaneously, the introduction of artificial intelligence (AI) brings new prospects for TEE image analysis and diagnostic support, significantly improving diagnostic accuracy and real-time decision-making capabilities. However, the application of TEE technology faces challenges, such as high costs, uneven technological diffusion, and high skill requirements for medical personnel. Therefore, establishing standardized training protocols and strengthening multidisciplinary collaboration is crucial. This paper reviews the application of TEE in cardiac surgery and its path toward educational and practical standardization from a global perspective, emphasizing its importance in improving the postoperative quality of life for patients and exploring future directions in technological innovation and educational optimization.

## Introduction

**Figure 1.**
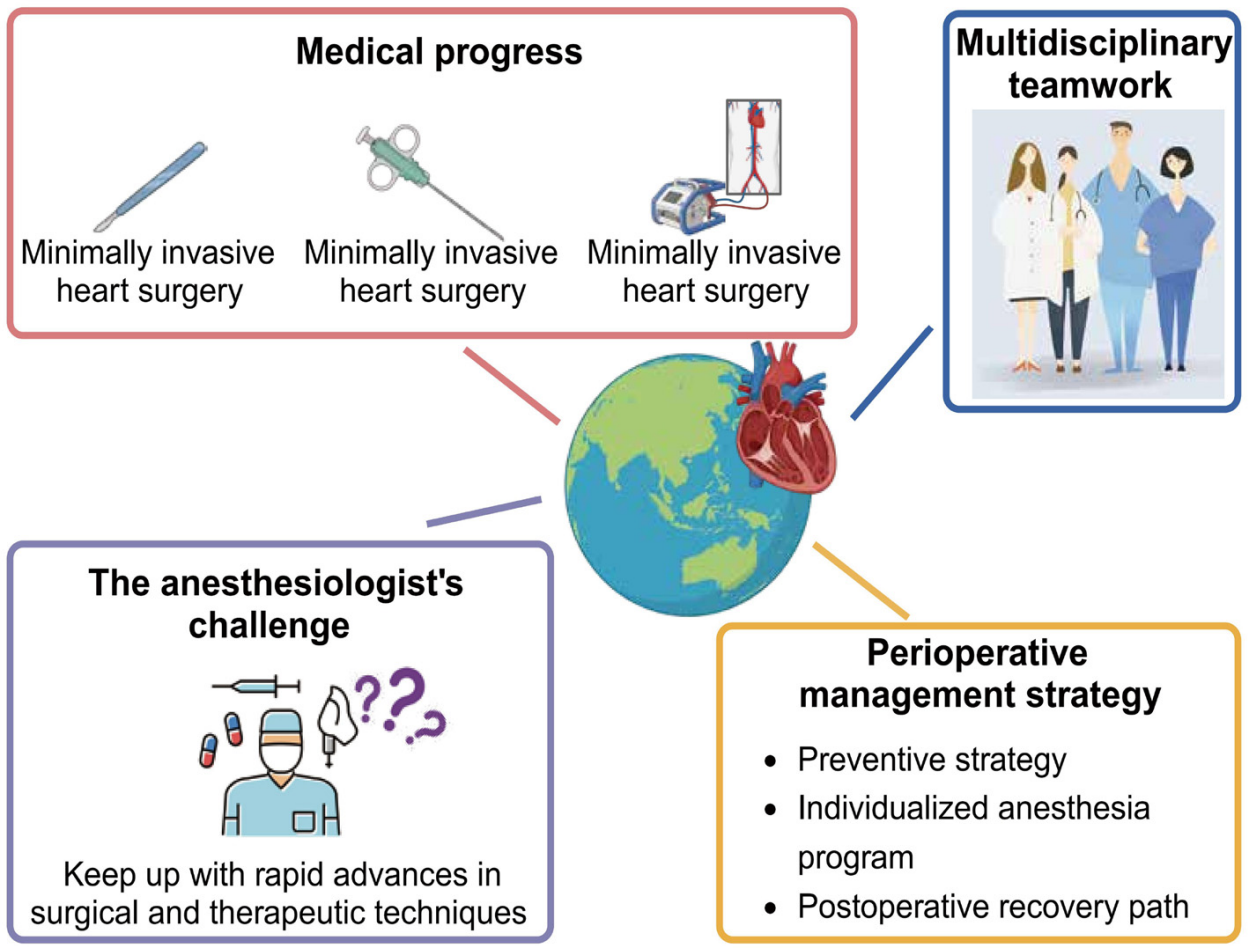
**Advances in the management of CVDs and the challenges faced by anesthesiologists**. CVD: Cardiovascular disease.

Cardiovascular diseases (CVDs) represent a global health challenge [1], continuing to attract widespread international attention and prompting extensive collaboration and research among healthcare professionals and researchers worldwide. Through these collective efforts, significant progress has been made in CVD management (Figure 1), particularly in minimally invasive cardiac surgery, interventional techniques, and innovative biocompatible cardiopulmonary bypass (CPB) circuits. However, these rapid advancements present new challenges for anesthesiologists, who must keep pace with evolving surgical and treatment techniques to ensure perioperative hemodynamic management and patient safety [2, 3].

Despite advances in the treatment and management of CVDs, heart disease has remained the leading cause of death in the United States since 1921. Although the mortality rate from CVD has decreased by 60% since 1950, recent years have seen an uptrend in both incidence and mortality rates due to factors, such as diabetes, an aging population, and health disparities [4]. This trend underscores the importance of continued focus and resource allocation in cardiovascular health. Additionally, with advancing age, individuals may become more prone to hypoventilation due to changes in respiratory drive, muscle strength, and chest wall compliance [5]. Goal-directed therapy (GDT) has demonstrated its superiority in reducing perioperative complications and hospital stays for patients undergoing high-risk surgeries, highlighting the significance of addressing cardiovascular insufficiency in perioperative management [6].

Advancements in medical technology, particularly the use of dynamic MRI, ultrasonography, and esophageal pressure measurement, have provided critical support for diagnosing and managing CVDs. However, these advancements introduce new challenges, such as the high costs associated with technological application, regional disparities in technology adoption rates, and the demand for highly skilled medical personnel [5]. Despite offering more stable and comprehensive data, the use of advanced hemodynamic monitoring technologies remains controversial regarding their invasiveness and frequency of monitoring [6].

The significance of anesthesia management in cardiac surgeries cannot be overlooked. Effective anesthesia management strategies can lower surgical risks, reduce postoperative complications, shorten hospital stays, and significantly improve patients’ quality of life [5, 6]. Appropriate anesthesia management is crucial for preventing postoperative functional recovery issues in patients with CVDs, and dynamic vascular compliance assessment, as an emerging indicator for predicting vascular reactivity, holds potential application value in anesthesia management [6].

Recent innovations in perioperative management strategies have significantly improved the safety and efficiency of surgeries, including the implementation of preventive strategies, the development of individualized anesthesia plans, and the application of enhanced recovery after surgery (ERAS) protocols [5, 6]. Additionally, research highlights the importance of interdisciplinary collaboration and management strategies tailored for specific populations, such as the homeless, in managing CVD. Patient-centered approaches are also emphasized for their role in improving quality of life and disease management outcomes [7, 8].

In summary, despite significant progress in treating and managing CVDs, the field continues to face evolving challenges. Future research and practice should explore new treatment modalities, optimize perioperative management strategies, and strengthen collaboration among multidisciplinary teams to further improve treatment outcomes and the quality of life for patients with CVDs.

### Innovations in transesophageal echocardiography (TEE) technology and its prospects in cardiac surgery

TEE has become a pivotal technology in the perioperative period of cardiac surgeries, ensuring both surgical success and patient safety. The development of cutting-edge technologies, such as novel probe designs and real-time three-dimensional (3D) TEE, has provided unprecedented accuracy and dynamic perspectives for surgical decision making. These advancements have been widely recognized for optimizing surgical outcomes and enhancing patient safety, marking a significant leap forward in the field [9].

**Table 1:** Applications and advantages of TEE in cardiac surgeries

**Category**	**Application**	**Advantages**
Hemodynamic assessment for cardio anesthesiologists	LVAD	Provides real-time left ventricular function assessment, guiding correct placement and operation of the device
	HTX (Heart transplant)	Assesses the function of the transplanted heart, monitoring postoperative complications
	ECMO (Extracorporeal membrane oxygenation)	Monitors heart and lung function, guiding the use of ECMO devices
Developing cardiac surgery procedures	Safety in seldinger peripheral cannulation for minimal invasive procedures	Enhances safety in peripheral cannulation for minimally invasive procedures
	Retrograde cardioplegia administration	Ensures effective delivery of retrograde cardioplegia, improving myocardial protection
Advanced monitoring	MV repair/implantation	Real-time monitoring of mitral valve repair or implantation, ensuring surgical success
	TV repair	Provides real-time imaging for tricuspid valve repair, guiding surgical procedures
	AV implantation, including futureless valves	Ensures correct placement of aortic valves, especially for futureless valves
Advanced navigation in structural procedures	TAVI	Provides real-time 3D views of cardiac structures, ensuring correct valve placement
	Mitraclip or triclip procedures	Provides precise navigation for valve repair, ensuring correct placement of devices
	Neochord	Guides placement of Neochord devices, ensuring procedural success
	LA occlusion	Ensures correct placement of left atrial occlusion devices, preventing strokes
	ASD amplatzer positioning	Provides real-time imaging, guiding correct placement of Amplatzer occluders
	PVL closure	Provides real-time navigation for paravalvular leak closure, ensuring surgical success
	Angiovac - percutaneous removal of pathological masses from the heart	Ensures safe and effective percutaneous removal of pathological masses from the heart

LVAD: Left ventricular assist device; HTX: Heart transplant; ECMO: Extracorporeal membrane oxygenation; MV: Mitral valve; TV: Tricuspid valve; AV: Aortic valve; TAVI: Transcatheter aortic valve implantation; LA: Left atrial; ASD: Atrial septal defect; PVL: Paravalvular leak.

The next generation of TEE probe technologies, featuring a specialized matrix array transducer design with 2500 independently operable piezoelectric crystals, has significantly enhanced image clarity and field of view while also improving patient comfort [10]. Additionally, the development of TEE simulators is crucial for training beginners, enabling them to practice in a stress-free environment and thereby enhancing their procedural skills [11]. Real-time 3D TEE technology represents another significant breakthrough in the evaluation of cardiac structures, particularly in the surgical planning for valvular heart diseases and congenital heart conditions. By offering stereoscopic images, clinicians can assess these structures from multiple angles and dimensions, which is especially valuable in patients with rheumatic heart disease (RHD). In such cases, 3D TEE provides more precise measurements of valvular areas and detailed anatomical structures [12]. With ongoing technological advancements, image clarity and resolution have significantly improved, and the application of automated image analysis techniques, such as automatic edge detection and machine learning algorithms, has proven valuable for rapid diagnosis and decision making. Innovations include automated 3D TEE left atrial appendage (LAA) analysis, which has demonstrated higher inter- and intra-observer reproducibility compared to manual analysis, thereby facilitating faster assessments [13]. However, studies evaluating the impact of staining standardization techniques on grading intracranial diseases (IDC) using convolutional neural networks (CNNs) found that staining standardization does not significantly influence classification outcomes, challenging the assumption that staining standardization enhances performance [14]. In selecting RHD patients for percutaneous balloon mitral valvuloplasty (PBMV), echocardiographic standards are critical to ensure the safety and success of interventions, highlighting the potential of integrating advanced imaging technologies in surgical planning and navigation. Interventional TEE, as part of structural catheter-based procedures, provides crucial guidance for cardiologists in positioning catheters and devices accurately [11, 12]. Furthermore, deep learning-based multi-network approaches for the automatic segmentation of cardiac structures in non-contrast-enhanced 3D MRI of the heart offer more accurate navigation for cardiac surgeries, showcasing the potential of automated image analysis to enhance both image clarity and the level of analysis automation [15].

**Figure 2.**
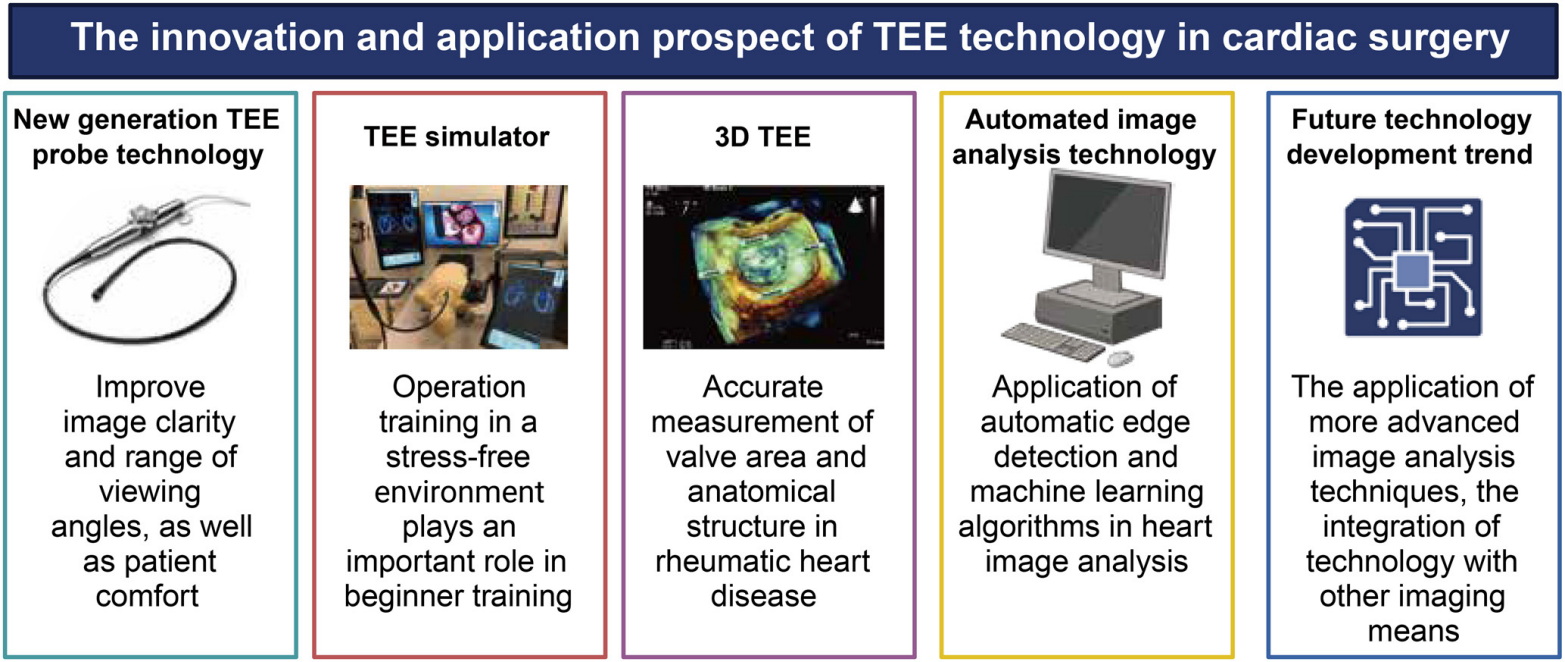
**The innovation and application prospect of TEE technology in cardiac surgery.** TEE: Transesophageal echocardiography.

To better illustrate the advantages of TEE in cardiac surgeries, Table 1 summarizes its applications and benefits across different procedures. With continuous technological advancement, the application of TEE in cardiac surgeries has become increasingly widespread and precise (Figure 2). Future development trends include the application of more advanced image analysis technologies and artificial intelligence (AI), along with further integration with other imaging technologies, offering enhanced support for decision making and execution in cardiac surgeries.

### The crucial role and advancements of TEE in cardiac surgeries

**Figure 3.**
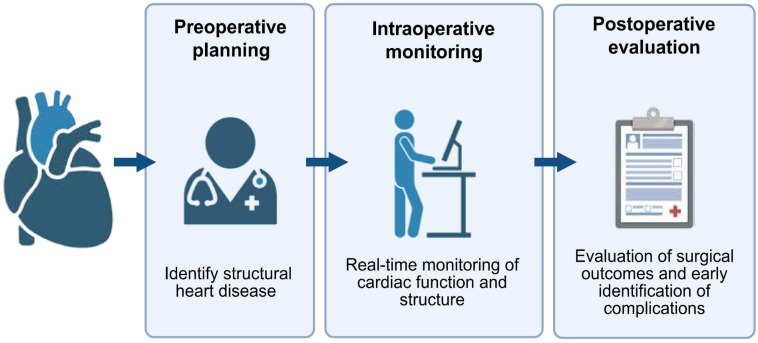
**The critical role and application of TEE in cardiac surgery.** TEE: Transesophageal echocardiography.

TEE plays a pivotal role in valvular and other structural cardiac surgeries, enhancing preoperative planning, detecting incidental anomalies requiring intraoperative attention, finely assessing postoperative and CPB outcomes, identifying related complications, and guiding surgical and anesthesia strategies to optimize patient outcomes [16] (Figure 3).

Since the incorporation of TEE into cardiac surgery, it has unveiled new clinical opportunities for anesthesiologists. Positioning the probe in the mid-esophagus of patients under general anesthesia not only meets procedural requirements but also improves patient comfort [17]. Anesthesiologists quickly embraced TEE to address the urgent need for rapid cardiac function assessment [18]. Although cardiologists have traditionally dominated the execution and interpretation of echocardiography, anesthesiologists have played a key role in monitoring vital signs during the perioperative period and in evaluating and managing cardiac complications [19]. Dr. Cahalan’s seminal review article in 1987 underscored the necessity for cardiac anesthesiologists to fully utilize imaging techniques in assessing the cardiovascular status of patients [20]. Dr. Cahalan significantly contributed to the development of perioperative TEE guidelines and advocated for cardiac anesthesiologists to be recognized as proficient echocardiographers [21–24]. These contributions greatly facilitated the integration of TEE in cardiac surgeries in the United States and laid a solid foundation for its global dissemination in cardiovascular anesthesia management [25].

The application of TEE in cardiac surgeries is comprehensive, playing an irreplaceable role in preoperative planning, intraoperative monitoring, and postoperative evaluation. The introduction of TEE has opened new clinical opportunities for anesthesiologists, allowing for the rapid and accurate assessment of cardiac function, which is crucial for surgical success and patient safety. In mitral valve repair surgeries, precise monitoring with 3D TEE enables surgeons to identify and correct residual mitral regurgitation due to inadequate annular reduction in real time, significantly reducing postoperative mitral regurgitation and improving cardiac function. The application of this technology has notably increased the success rates of surgeries and delivered superior therapeutic outcomes for patients [26]. For the closure of atrial septal defects (ASD), using TEE as a real-time navigation tool has successfully guided the precise placement and release of occluders. High-resolution imaging with TEE allows for accurate measurement of the defect size and selection of an appropriate occluder, directly ensuring surgical success and confirming the correct position of the occluder with no residual shunt in postoperative assessments [27]. Transcatheter aortic valve replacement (TAVR) exemplifies another area where the value of TEE is prominently displayed. In TAVR procedures, 3D TEE provides real time, 3D views of cardiac structures, assisting in confirming the proper placement of artificial valves and rapidly identifying potential complications, such as aortic regurgitation. This information is critical for the success of the surgery and helps reduce the need for contrast agents, which is especially beneficial for patients at risk of renal disease [28–30]. In LAA closure (LAAC) procedures using the WATCHMAN FLX device, TEE plays a crucial role in assessing closure effectiveness. This technology shows great potential in enhancing surgical success rates, reducing postoperative complications, and providing patients with an effective treatment option to prevent stroke [31]. To better illustrate the advantages of TEE in cardiac surgeries, Table 1 summarizes its applications and benefits across different procedures.

Overall, the application of TEE has significantly enhanced the safety and success rates of cardiac surgeries. TEE provides indispensable imaging support in various procedures, including mitral valve repair, ASD closure, TAVR, and LAAC, ensuring the successful execution of surgeries and patients’ postoperative recovery. With the continuous introduction of next-generation devices, the use of TEE in cardiac surgeries is expected to become even more widespread and precise, further elevating the safety and success rates of these procedures.

### Application and advantages of TEE in pediatric cardiac surgery

In recent years, TEE has seen increased use in pediatric cardiac surgery. It provides real-time imaging and hemodynamic monitoring, which are crucial for intraoperative management and postoperative evaluation. TEE is a powerful diagnostic and monitoring tool that can significantly improve the outcomes of pediatric cardiac surgeries by providing valuable real-time data during operations.

Firstly, the application of TEE in preoperative planning and intraoperative real-time assessment offers a major advantage in pediatric cardiac surgery. TEE allows doctors to understand the anatomical structure and functional state of the heart in detail, facilitating more precise surgical planning. During surgery, TEE helps identify and address various cardiac abnormalities, such as cardiac insufficiency and residual lesions, thereby significantly improving the safety and success rate of operations [32].

TEE also excels in the evaluation and management of heart valve diseases. For pediatric patients with valvular heart disease, TEE provides precise valve function assessments and real-time monitoring of dynamic changes in the valves. For example, in patients with aortic stenosis, TEE can help evaluate the severity of the stenosis and guide anesthetic management and hemodynamic support during surgery, optimizing intraoperative management [33].

Moreover, TEE plays an important role in predicting postoperative fluid responsiveness and guiding fluid management. By evaluating dynamic parameters such as changes in arterial blood flow velocity and left ventricular velocity-time integrals, TEE can effectively predict fluid load response in mechanically ventilated children, optimizing postoperative fluid management [34].

In complex surgeries, such as ventricular septal defect closure, TEE is crucial. It enables precise localization of the defect and real-time evaluation of the closure effect, optimizing the placement of surgical instruments. This not only increases the success rate of surgeries but also reduces operation time and postoperative complications [35].

The integration of TEE with fluoroscopic imaging shows promise in pediatric cardiac catheterization procedures. This technology’s safety and accuracy have been validated in several studies, providing a new imaging guidance method for complex surgeries, making them more precise and safer [36].

In evaluating cardiovascular coupling after pediatric heart surgery, TEE provides valuable data. Studies have shown that cardiovascular coupling values in children after heart surgery are typically high (above 1), which is significant for assessing cardiac function and optimizing treatment strategies [37].

Finally, TEE is crucial for real-time monitoring of acute cardiac events during surgery. In non-cardiac surgeries, especially in high-risk patients, acute cardiac events such as myocardial ischemia or pericardial effusion may occur. TEE allows for real-time cardiac function monitoring, enabling timely detection and management of these emergencies, thus enhancing intraoperative safety and patient prognosis [38].

In summary, TEE in pediatric cardiac surgery not only enhances the safety and efficacy of operations but also provides crucial real-time imaging support for intraoperative and postoperative management. Its extensive application in preoperative planning, intraoperative assessment, postoperative management, and prediction of postoperative responsiveness significantly improves overall outcomes in pediatric cardiac surgery. Therefore, it is recommended to widely use TEE in pediatric cardiac surgeries to further improve surgical and recovery outcomes for pediatric patients.

### Application of TEE in non-cardiac surgery

In recent years, TEE has not only played a vital role in cardiac surgeries but has also shown significant value in non-cardiac surgeries, particularly in managing patients with cardiac diseases. In these patients, TEE provides real-time hemodynamic monitoring, helping doctors better assess and manage cardiac function during surgery, thereby improving surgical safety and success rates.

The introduction of TEE into non-cardiac surgery has unveiled new clinical opportunities for anesthesiologists. TEE can provide real-time assessments of left and right ventricular function, assisting doctors in identifying and managing potential cardiac insufficiency during surgery. For example, in abdominal surgeries, intraoperative bleeding or improper fluid management may increase cardiac load. By monitoring cardiac function with TEE, doctors can adjust fluid therapy in real time to ensure hemodynamic stability [39].

TEE is also crucial in evaluating and managing heart valve diseases. In patients undergoing non-cardiac surgeries, especially those with valvular heart disease, TEE can help assess valve function and monitor dynamic changes during surgery. For instance, in patients with aortic stenosis, TEE can evaluate the severity of the stenosis and guide intraoperative anesthetic management and hemodynamic support [33].

Additionally, TEE can monitor acute cardiac events, such as myocardial ischemia or pericardial effusion, that may occur during surgery. In non-cardiac surgeries, especially in high-risk patients, acute cardiac events may occur. TEE allows for real-time cardiac function monitoring, enabling doctors to detect and manage these emergencies promptly, thereby improving intraoperative safety and prognosis [38].

In non-cardiac surgeries, TEE also guides fluid management and assesses hemodynamic status. For example, in liver resection surgeries, TEE can monitor the compression of the inferior vena cava and hepatic veins, helping doctors better manage hemodynamics during the procedure [40].

In conclusion, the application of TEE in non-cardiac surgeries provides crucial hemodynamic information and guides intraoperative management, enhancing surgical safety and success rates. Therefore, it is recommended to widely apply TEE in non-cardiac surgeries, especially in patients with cardiac conditions.

### Application of AI in TEE

In recent years, the application of AI in medical imaging has gradually emerged, especially in TEE. AI technology shows great potential in image analysis, diagnostic accuracy, and real-time decision support, bringing new prospects for the clinical application of TEE.

AI algorithms can significantly enhance TEE image processing and analysis capabilities, particularly in the automated recognition and measurement of complex cardiac structures. For example, a study developed AI-based semi-automated software for the dynamic geometric analysis of the tricuspid valve. This software analyzes valve morphology and dynamic changes throughout the cardiac cycle, providing highly reproducible data analysis, reducing the need for manual intervention, and improving diagnostic accuracy [41]. Additionally, AI has shown significant advantages in the automated evaluation of left ventricular function. A study utilized AI technology to automatically measure the mitral annular plane systolic excursion (MAPSE), achieving rapid and quantitative left ventricular function assessment, showing potential for application in critically ill patients [42].

Through deep learning algorithms, AI learns from large amounts of TEE image data, enabling the identification of potential cardiac lesions and providing accurate diagnostic recommendations. For example, the AI model LAT-AI was developed to predict the presence of LAA thrombosis using clinical and transthoracic echocardiographic features, with predictive accuracy superior to traditional left ventricular ejection fraction and CHA2DS2-VASc scores [43]. Another study demonstrated AI’s application in the automatic detection and measurement of aortic annulus size, with results highly consistent with CT measurements, showcasing AI’s immense potential in improving diagnostic consistency and accuracy [44].

Intraoperatively, AI systems can analyze TEE image data in real time, providing immediate diagnostic advice and decision support to help doctors quickly manage intraoperative emergencies. For example, the automated MAPSE measurement tool (autoMAPSE) can perform measurements in nearly every set of cardiac chamber views in less than a second, aiding in real-time monitoring of left ventricular function changes during surgery [42]. In critically ill patients’ post-cardiac surgery, AI tools for continuous monitoring of left ventricular function exhibit high accuracy and trend recognition capabilities, crucial for timely treatment strategy adjustments [45]. Additionally, a study demonstrated the application of AI-driven TEE in complex interventional procedures, enhancing surgical precision and safety through real-time image analysis and navigation [46].

Despite the promising prospects of AI in TEE, clinical practice still faces challenges. The training of AI models relies on high-quality annotated data, and the diversity and quality of the data directly affect the model’s generalization ability. Moreover, AI systems require rigorous clinical validation and regulation to ensure their reliability and safety in different clinical scenarios [47]. In the future, as AI technology and TEE equipment continue to advance, AI is expected to play an increasingly important role in improving diagnostic efficiency, reducing intraoperative complications, and optimizing treatment plans.

In summary, AI has broad application prospects in TEE. By introducing AI technology, the clinical application of TEE can be significantly enhanced, providing more precise image analysis and diagnostic support, thereby further improving patient surgical and recovery outcomes.

### The path to education and standardization of TEE practices from a global perspective

Anesthesiologists play a crucial role in applying TEE during the perioperative period. TEE technology offers anesthesiologists a precise and comprehensive method for assessing and monitoring cardiac structures and functions, which is particularly important during surgeries. As the technology has evolved and specialization has deepened, the use of TEE has expanded from cardiologists and ultrasound experts to anesthesiologists, especially those specializing in cardiac anesthesia [48]. This shift is driven by anesthesiologists’ profound understanding of cardiovascular physiology in the operating room and the need to apply TEE directly for cardiac emergency monitoring [49, 50].

Despite the critical importance of TEE during the perioperative period for patient care, significant disparities exist in TEE training and practice among different medical institutions and regions. These discrepancies underline the urgent need for uniform training content and assessment standards to enhance the overall quality of TEE practice. Perioperative TEE often requires collaboration between two specialized nursing staff and a dedicated cardiac ultrasonographer. However, in practice, anesthesiologists frequently need to perform TEE independently without immediate support from cardiologists or ultrasound experts [51–53].

Globally, TEE training models exhibit diversity. In the United States, guidelines for intraoperative echocardiography are primarily established by the American Society of Anesthesiologists (ASA), the Society of Cardiovascular Anesthesiologists (SCA), and the American Society of Echocardiography (ASE). The National Board of Echocardiography (NBE) serves as the primary certifying body, offering a pathway for TEE competency certification through training and examination since 1998 [54]. In contrast, TEE training and certification standards vary across other countries and regions, such as China, India, and Europe, highlighting the necessity of establishing international or at least regional unified standards.

**Figure 4.**
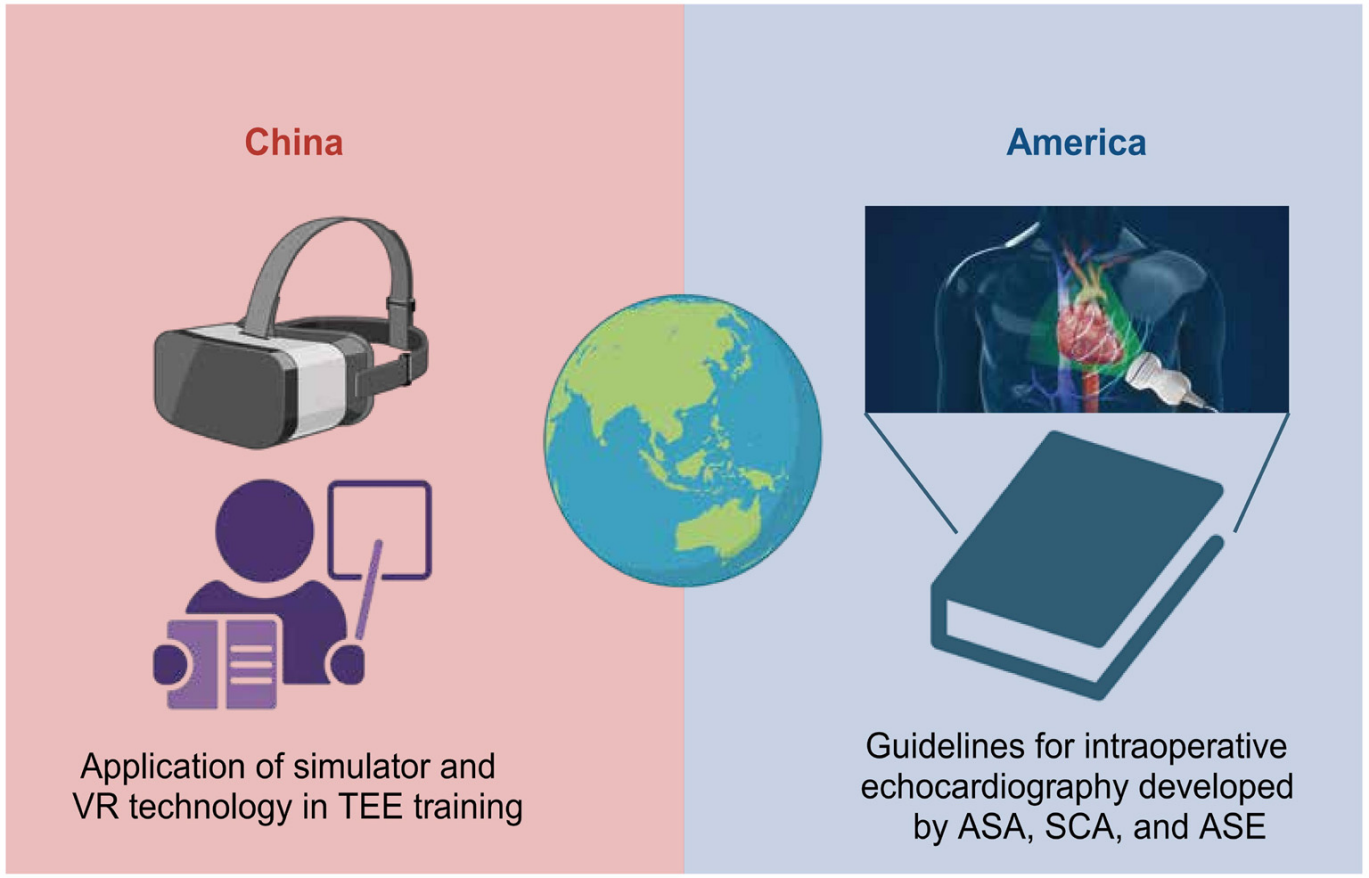
**A global perspective on the standardization of TEE education and practice.** TEE: Transesophageal echocardiography; VR: Virtual reality; ASA: The American Society of Anesthesiologists; SCA: Society of Cardiovascular Anesthesiologists; ASE: The American Society of Echocardiography.

Particularly in China, despite significant progress in TEE technology, training and certification for TEE examinations are still lacking for many physicians. Only a minority of anesthesiologists have undergone formal TEE training, with most training durations not exceeding one month [55]. Moreover, the high degree of specialization in ultrasonography and the requirements for the National Color Doppler Flow Imaging (CDFI) certification limit anesthesiologists’ capacity to issue TEE reports, relegating TEE more as a monitoring tool than a formal diagnostic method in practice [55] (Figure 4).

**Figure 5.**
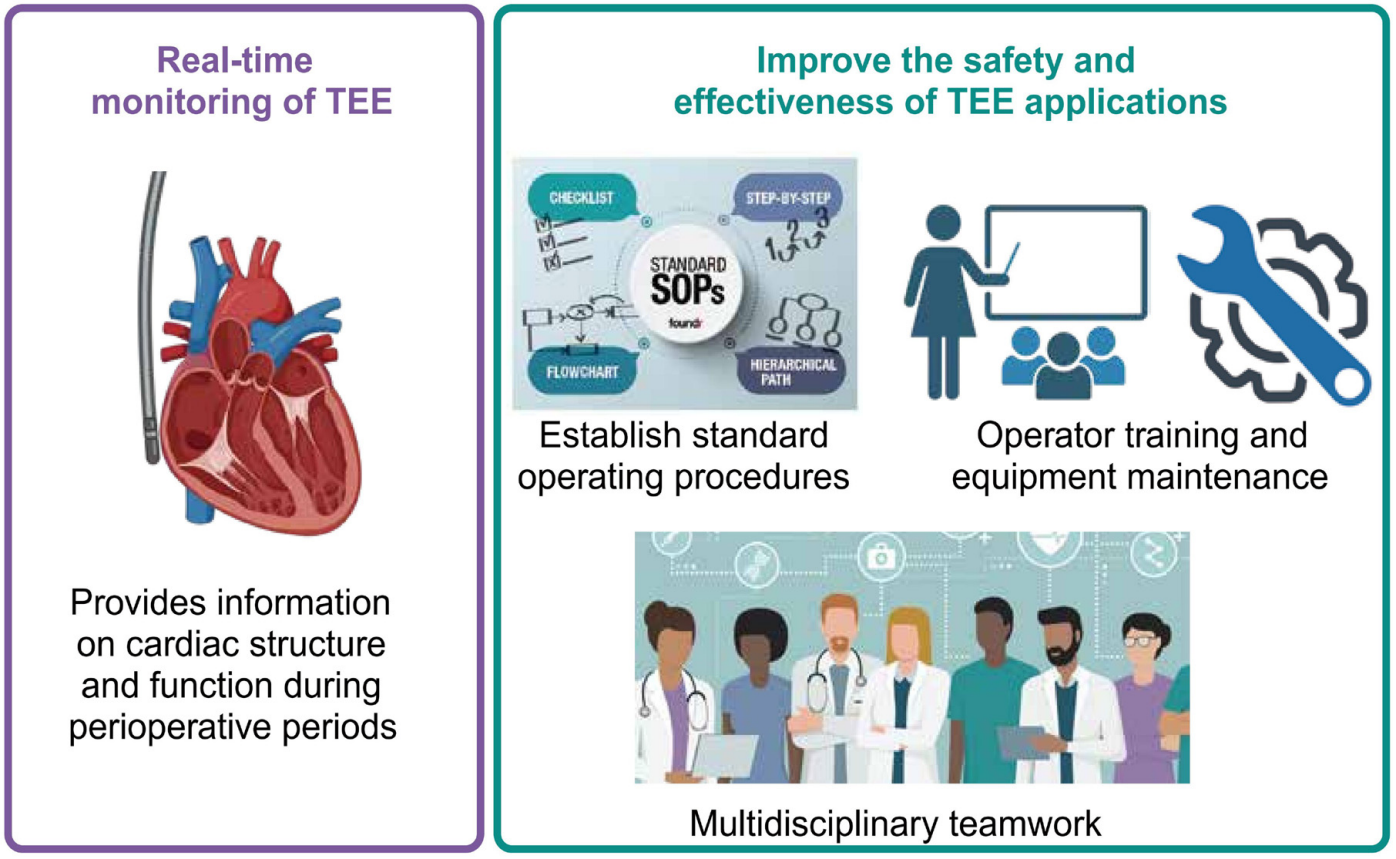
**The complexity and quality control strategies of perioperative TEE application.** TEE: Transesophageal echocardiography.

Addressing these issues and formulating uniform training content and assessment standards are particularly important. These standards should encompass the fundamentals of TEE, operational skills, clinical applications, management of complications, and accurate interpretation of TEE findings. Using simulators and virtual reality (VR) technology could accelerate the learning curve while enhancing safety and efficiency in learning [56]. For instance, pioneers in China’s field of cardiovascular anesthesia have begun to explore methods for standardizing perioperative TEE techniques and enhancing patient care standards. The establishment of the Chinese Society of Cardiothoracic and Vascular Anesthesiology (CSCVA) and its subsidiary training centers for cardiovascular anesthesiologists reflect efforts toward uniform standards [57, 58]. Additionally, the TEE training alliance at West China Hospital, Sichuan University, and its successful practice of visualized perioperative ultrasonography education offer a replicable model for other institutions [59, 60].

In summary, the standardization of perioperative TEE training is critical to enhancing medical quality and patient safety. Establishing uniform training content and assessment standards ensures that anesthesiologists, regardless of their country or region, can perform TEE at the same professional level, thus promoting consistency and high quality in global medical practice.

### The complexity of TEE application, quality control, and standardization strategies

TEE is an indispensable real-time observational tool during the perioperative period, providing essential information on cardiac structures and functions to guide immediate decisions in cardiac surgeries. However, the application of TEE is challenged by the inherent complexities and dynamic changes at various stages of cardiac surgeries, particularly around the use of CPB. During cardiac surgeries, especially before and after CPB, there are diverse diagnostic requirements for the heart’s walls, chambers, valves, and blood flow, posing challenges in acquiring quantitative hemodynamic data [57]. Before CPB, physicians must navigate challenges associated with hemodynamic fluctuations, electrocautery interference, positive pressure ventilation, fluid shifts, and surgical manipulations. After CPB, issues such as myocardial ischemia-reperfusion injury, vasoactive medications, cardiac assist devices, and arrhythmias also impact the assessment with TEE [57].

Immediate postoperative TEE assessments are particularly challenging yet crucial for swiftly evaluating the quality of surgical outcomes, identifying potential complications, and assisting cardiac surgeons in making decisions regarding the use of CPB. An urgent TEE assessment may be necessary in cases of severe hemodynamic instability. Even if a cardiac or ultrasound physician outside the operating room has been notified in advance, patients may still face complex and unpredictable risks while waiting for the evaluation [61].

Regarding quality control and standardization, studies indicate that higher staffing levels of care are associated with improved care processes and patient outcomes. These improvements include a reduction in the proportion of patients who leave without being seen (LWBS), decreased time to treatment, increased patient satisfaction, and shorter waiting times [62]. However, research on the relationship between emergency department staffing levels and the quality of care is relatively limited.

Establishing standard operating procedures (SOPs) is fundamental to ensuring the quality of TEE operations. SOPs should encompass all aspects of TEE, including patient assessment, equipment preparation, operational techniques, image acquisition, data interpretation, and report generation. Additionally, SOPs should outline the management of potential complications and provide specific guidance for the immediate postoperative assessment of cardiac function and surgical outcomes. Human errors can be minimized by adopting a multidisciplinary team approach involving anesthesiologists, cardiac surgeons, cardiac ultrasonographers, and the nursing team. This collaborative effort ensures a comprehensive evaluation of TEE data from various professional perspectives, enhancing decision-making accuracy [63].

Ultimately, implementing quality control measures and adhering to SOPs can significantly enhance patient safety during the perioperative use of TEE. This includes ensuring the correct use and maintenance of equipment, the professional competence of operators, and the accurate assessment of patients’ cardiac conditions. Rapid and precise TEE assessments are crucial for guiding clinical decisions and preventing potential complications [63]. Therefore, quality control and standardization are vital for the practical and safe application of perioperative TEE, ensuring that medical teams can follow best practice guidelines to minimize the risks patients face during surgery (Figure 5).

### Innovations and challenges of TEE: A global perspective and future outlook

TEE has become an integral component of cardiac surgeries during the perioperative period, with its extensive application in clinical anesthesia prompting anesthesiologists to delve into diagnostic and therapeutic areas traditionally beyond their scope. The role of anesthesiologists has evolved from merely being service providers to decision-making architects, now assuming the role of “TEE physicians” during the perioperative period and significantly influencing clinical outcomes [64]. Studies have shown that using TEE reveals new cardiodynamic findings in 20%–33% of cases, leading to changes in surgical management in 10%–25% of cases [65, 66]. However, despite the advocacy for systematic and comprehensive TEE assessments, the complexity of TEE operations has gradually increased due to patient-centered surgeries and the dynamics of time-sensitive clinical decision making [67].

**Figure 6.**
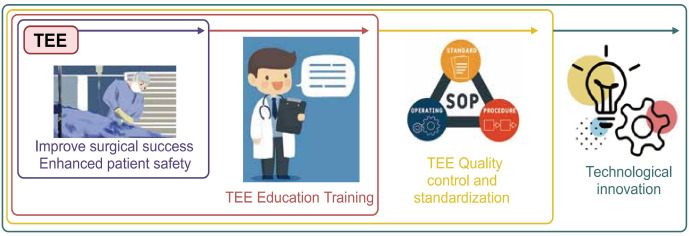
**Comprehensive analysis and outlook on perioperative TEE technology.** TEE: Transesophageal echocardiography.

Fyfe highlighted the dual responsibilities of anesthesiologists and TEE interpreters during surgery, which could impede their effectiveness in either role [68]. Recent systematic reviews have shown an 84% concordance rate in TEE interpretation between cardiac anesthesiologists and novice echocardiography technicians [69], indicating that despite inherent limitations and the dated nature of the data, a high degree of consistency in TEE interpretation between cardiac anesthesiologists and echocardiography technicians may still be revealed by modern studies. Furthermore, multiple studies have indicated that continuous quality improvement (CQE), operator mentoring, clinical insight, and the depth of focus on TEE are more important than the professional background of the operator [70–72].

With the advancement of surgical techniques and the increasing complexity of patients’ pathophysiological characteristics, perioperative TEE has become a cornerstone in cardiac surgeries, spanning over 40 years. Anesthesiologists who receive specialized TEE training serve as “echocardiography experts” during surgeries. Their ongoing acquisition of skills and expertise aims to provide surgeons with timely and accurate information, thus facilitating proficient perioperative patient management [73]. Despite this, our understanding of the utilization patterns of TEE across different global institutions still needs to be improved.

Historical surveys from 1995 to 2014 have highlighted significant differences in the application and management of TEE across various countries and regions. Surveys from the United States, Canada, the United Kingdom, and Spain revealed the widespread use of TEE in cardiac surgeries and the pivotal role played by anesthesiologists [19, 49, 74–80]. In contrast, Russia has limited and isolated applications of TEE, with only a few cardiac surgery hospitals integrating intraoperative TEE into their anesthesia protocols [81, 82]. Surveys from India and China reflect the utilization and challenges of TEE in cardiac anesthesia [55, 83–86].

The advancement of TEE technology will depend on overcoming its operational and interpretative complexities, strengthening multidisciplinary team collaboration, and continually improving training and education. Innovations in education and training, such as using TEE simulators, may enhance training efficiency and quality. Additionally, with technological advancements and changes in surgical conditions, the application of TEE is expected to expand further, including monitoring and managing critical hemodynamic fluctuations in non-cardiac surgeries [77, 78]. Moreover, with globalization and the development of information technology, TEE training and application are moving toward internationalization and standardization, promoting TEE’s global adoption and standardized practices. Efforts by the Chinese Medical Doctor Association (CMDA) in TEE training and application and proposals to integrate essential TEE guidance into medical education indicate the standardization and widespread adoption of TEE in China and globally [87]. Finally, technological innovations, such as using TEE simulators, will further enhance the efficiency and quality of TEE training, supporting the capabilities of cardiac anesthesiologists and other medical professionals in perioperative management [87]. Through these efforts, TEE will continue to play an indispensable role in cardiac surgeries and beyond, offering improved perioperative management for patients and enhancing clinical outcomes.

## Conclusion

This study provides a comprehensive analysis of the application of TEE in cardiac surgeries during the perioperative period, focusing on education, training, quality control, standardization, and the challenges faced along with future directions for development (Figure 6). Drawing from an in-depth examination of the integration of TEE into clinical anesthesia practices in China, the following conclusions are drawn.

Firstly, TEE has become an indispensable technology in cardiac surgeries, crucial in enhancing surgical success rates and patient safety. Advances in probe technologies and real-time 3D imaging have provided unprecedented precision and dynamic perspectives for surgical decision making. However, these technological advancements have also introduced new challenges, including high costs, regional disparities in adopting the technology, and increased demands on medical personnel.

Secondly, while the importance of TEE technology to anesthesiology is increasingly recognized, the standardization of TEE training and implementation in China still faces significant obstacles. These include uneven resource allocation, training gaps, and issues related to workload intensity [55, 85, 88, 89]. Inequalities in regional medical resources, conservative cultural norms, and anesthesiologists’ lack of experience in cardiac anesthesia further limit the widespread adoption and application of TEE technology.

Furthermore, the quality of TEE training in China heavily relies on the specific circumstances of individual hospitals. Most anesthesia residency training programs lack adequate TEE content, underscoring the necessity for systematic and standardized education and training [90, 91]. Addressing this issue requires establishing unified training standards and assessment systems, employing multidisciplinary team collaboration, and implementing quality control measures and SOPs as critical strategies to enhance the effectiveness of TEE application during the perioperative period.

Facing the challenges of an aging population and the rapid increase in the number of cardiac surgeries, cardiovascular anesthesiologists are expected to experience increased workload intensity, presenting challenges related to professional burnout, investment in career development, and issues of medical violence [85, 89]. Therefore, the future development of TEE technology will depend on technological innovation, optimization of education and training, strengthening of quality control and standardization, and deeper multidisciplinary team collaboration. The application of TEE technology in cardiac surgeries has significantly improved surgical safety and success rates. The future development and application of TEE technology will require continued technological innovation, standardized education and training, quality control, and interdisciplinary collaboration to further enhance clinical outcomes in cardiac surgeries, providing patients with better prognoses. Addressing the unique challenges in developing regions like China through targeted solutions and measures is critical to achieving widespread adoption and application of TEE technology.

## Data Availability

The data that support the findings of this study are available from the corresponding author upon reasonable request.
